# Next-generation molecular diagnostics: Leveraging digital technologies to enhance multiplexing in real-time PCR

**DOI:** 10.1016/j.trac.2023.116963

**Published:** 2023-02-09

**Authors:** Louis Kreitmann, Luca Miglietta, Ke Xu, Kenny Malpartida-Cardenas, Giselle D’Souza, Myrsini Kaforou, Karen Brengel-Pesce, Laurent Drazek, Alison Holmes, Jesus Rodriguez-Manzano

**Affiliations:** a Department of Infectious Disease, Faculty of Medicine, Imperial College London, UK; b Research & Development, BioMérieux S.A, Marcy-l’Etoile, France; c Department of Electrical and Electronic Engineering, Faculty of Engineering, Imperial College, London, UK

**Keywords:** Real-time polymerase chain reaction, Machine learning, Amplification curve analysis, Melting curve analysis, Nucleic acid amplification techniques, Molecular diagnostics

## Abstract

Real-time polymerase chain reaction (qPCR) enables accurate detection and quantification of nucleic acids and has become a fundamental tool in biological sciences, bioengineering and medicine. By combining multiple primer sets in one reaction, it is possible to detect several DNA or RNA targets simultaneously, a process called multiplex PCR (mPCR) which is key to attaining optimal throughput, cost-effectiveness and efficiency in molecular diagnostics, particularly in infectious diseases. Multiple solutions have been devised to increase multiplexing in qPCR, including *single-well* techniques, using target-specific fluorescent oligonucleotide probes, and *spatial multiplexing,* where segregation of the sample enables parallel amplification of multiple targets. However, these solutions are mostly limited to three or four targets, or highly sophisticated and expensive instrumentation. There is a need for innovations that will push forward the multiplexing field in qPCR, enabling for a next generation of diagnostic tools which could accommodate high throughput in an affordable manner.

To this end, the use of machine learning (ML) algorithms (data-driven solutions) has recently emerged to leverage information contained in amplification and melting curves (AC and MC, respectively) – two of the most standard bio-signals emitted during qPCR – for accurate classification of multiple nucleic acid targets in a single reaction. Therefore, this review aims to demonstrate and illustrate that data-driven solutions can be successfully coupled with state-of-the-art and common qPCR platforms using a variety of amplification chemistries to enhance multiplexing in qPCR.

Further, because both ACs and MCs can be predicted from sequence data using thermodynamic databases, it has also become possible to use computer simulation to rationalize and optimize the design of mPCR assays where target detection is supported by data-driven technologies. Thus, this review also discusses recent work converging towards the development of an end-to-end framework where knowledge-based and data-driven software solutions are integrated to streamline assay design, and increase the accuracy of target detection and quantification in the multiplex setting. We envision that concerted efforts by academic and industry scientists will help advance these technologies, to a point where they become mature and robust enough to bring about major improvements in the detection of nucleic acids across many fields.

## 1 Introduction

Accurate detection and quantification of nucleic acids is a fundamental procedure in life sciences research, bioengineering, and diagnostics. In clinical microbiology, standard culture often remains the gold-standard but suffers from numerous limitations (such as low turnaround time and sensitivity), and nucleic acid amplification tests (NAATs) have brought about major progress by providing fast, affordable and accurate means to detect pathogens and antimicrobial resistance genes in clinical samples [1]. Amongst NAATs, the polymerase chain reaction (PCR) has become one of the most standard tools in biological sciences because of its large dynamic range, high sensitivity and reproducibility [2]. In its most basic form, PCR uses thermal cycling and a pair of specific oligonucleotides (primers) to target the action of a thermostable DNA polymerase to obtain millions to billions of copies of a given DNA template [2]. Beyond mere detection, it is often important to quantify precisely the amount of template DNA present at the beginning of the reaction, which can be achieved by monitoring the concentration of amplicons at each cycle [3], a process named real-time (or quantitative) PCR (qPCR).

In most clinical applications, it is important to detect several DNA targets simultaneously by combining multiple sets of primers into one reaction, a process called multiplex PCR (mPCR) that is key to reducing the time and cost of the reaction. Furthermore, because dozens of different pathogens can be responsible for similar clinical manifestations, their concomitant detection in limited amounts of patient samples can be both an important clinical endpoint and a technical challenge [4]. Much effort and ingenuity have been invested to devise both *single-well* and *spatial multiplexing* solutions to improve multiplexing capabilities of PCR-based assays. Single-well techniques rely on the use of target-specific fluorescent oligonucleotide probes emitting at different wavelengths (enabling target identification through a specific color-sequence mapping), or on post-PCR processes. Spatial multiplexing techniques leverage microfluidics technologies for spatial segregation of PCR, allowing for parallel yet spatially distinct amplification and detection of different targets. However, these solutions have intrinsic limitations, including their relatively high cost, low throughput and the requirement for specialist equipment, which make them suboptimal in clinical applications (detailed in Table 1).

In recent years, technological innovations in the fields of computer science and artificial intelligence (AI) have made it possible to convert the data that surround us into meaningful information, and subsequently, informed decisions. However, the use of machine learning (ML) algorithms (‘software’ or data-driven solutions) to extract information from amplification reactions has been fairly unexplored, and PCR analysis still relies on rudimentary data processing methods. As a result, valuable information – present in most diagnostic platforms – that could be used to enhance assay performance is discarded, compromising time, overall cost and patient outcomes. However, much progress has been made recently at the intersection of ML and molecular biology to leverage information contained in amplification and melting curves (AC and MC, respectively) – two of the most standard bio-signals emitted during qPCR – for accurate classification of multiple DNA targets in a single reaction. Thus, our main objective is to demonstrate and illustrate that data-driven solutions can be coupled with commonly used *single-well* and *spatial multiplexing* solutions to increase multiplexing in PCR, leading to improved molecular diagnostic assays, without the need to change hardware or reaction chemistry. In the first part of this review, we will provide a concise description of existing data-driven technologies aiming to enhance multiplexing capabilities of qPCR assays, describe in more detail their most recent developments and offer some insights into future research efforts. Most application examples that we provide throughout the text are related to molecular diagnostics, especially in medical microbiology and infectious diseases, but it is important to note that these technologies can be applied across all disciplines where accurate detection and quantification of nucleic acids is critical.

Despite its apparent complexity, a multiplex PCR mix contains oligonucleotides whose physical properties are governed by the laws of thermodynamics. Knowledge-based software have been developed to compute secondary structures, interactions and degree of hybridization (as a function of temperature) of DNA molecules [5–9]. Furthermore, in the multiplex setting, the presence of multiple primers in one reaction increases the chances of obtaining spurious amplification products (e.g., primer dimers), which complicates the design of multiplex assays (i.e. the selection of optimal primer sets). Thus, in the last part of this review, we will also describe software solutions designed to anticipate unwanted interactions and predict the output of amplification reactions, and illustrate how they can be used to rationalize and optimize the design of mPCR assays where target detection is supported by data-driven technologies. We will highlight recent work converging towards the development of an end-to-end framework, where knowledge-based and data-driven software solutions are integrated to streamline assay design and increase the accuracy of target detection and quantification in the multiplex setting. We envision that concerted efforts by academic and industry scientists will help advance these technologies, to a point where they become mature and robust enough to bring about major improvements in the detection of nucleic acid across many fields.

## 2 Existing technologies in multiplex real-time PCR

**Fig. 1:**
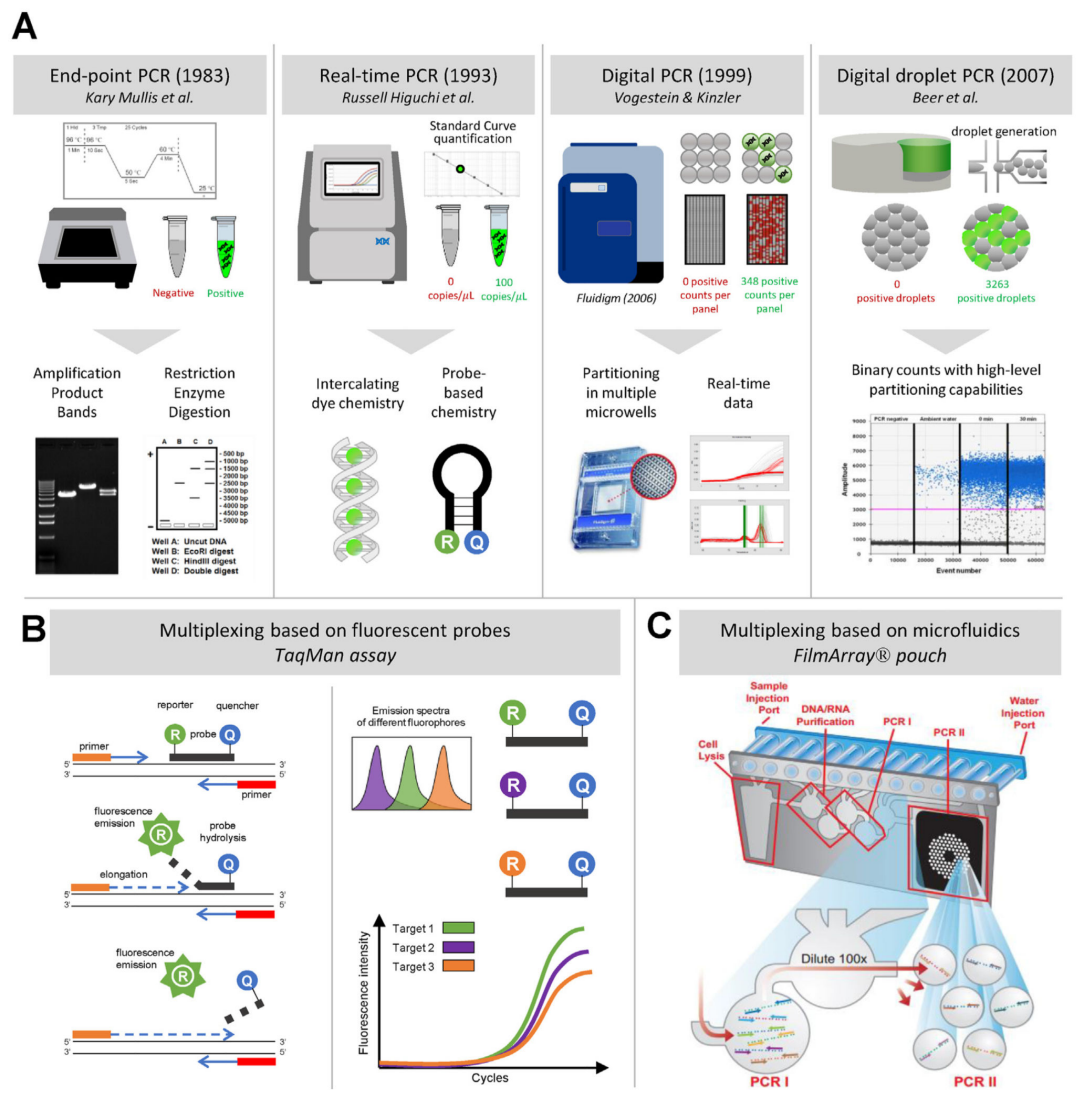
Existing PCR chemistries and instruments A. Historical overview of the development of PCR techniques, including real-time PCR using non-specific intercalating dyes or target-specific fluorophore-coupled oligonucleotide probes, and digital PCR using end-point or real-time fluorescent measurement. B. One simple technique for single-well multiplexing in PCR is using fluorescent probes (presented here are TaqMan probes). Because probes are sequence-specific, detection of fluorescence can be used for target identification. The main limitation of this technique is that only a limited set of colors can be detected in one reaction (up to 6), prompting the development for alternate means of obtaining high-level multiplexing. C. Spatial multiplexing: microfluidics-based platforms allow for parallel yet spatially distinct amplification of multiple targets. Some platforms are FDA-cleared and used increasingly in diagnostic microbiology (presented here is a FilmArray® pouch).

The first challenge encountered when transitioning from singleplex to mPCR is amplicon detection, i.e. devising methods that can specifically detect each amplification in a mixture of all possible PCR end-products. Among the most ancient of such techniques is gel electrophoresis, where amplicons migrate on a gel matrix under an electric current and are separated according to their size. This can be coupled with restriction length fragment polymorphism (RLFP) analysis (Fig. 1A).

In qPCR, monitoring the concentration of amplicons at each cycle enables to precisely quantify the amount of template DNA present at the beginning of the reaction [3]. This can be achieved using non-specific fluorescent intercalating dyes, that emit light upon interaction with double-stranded DNA, or sequence-specific oligonucleotide probes, which permit detection after hybridization of the probe with its complementary sequence. Several variations of such probes have been designed [10], such as TaqMan [11], molecular beacons [12], Scorpion [13] and Sunrise primers [14]. Probes offer a convenient tool for multiplexing because they can be coupled with different fluorophores (up to 6 colors), and this color-sequence correspondence is useful to monitor the real-time amplification of different targets in the multiplex setting (Fig. 1B).

Another important challenge in mPCR is assay design, because the presence of multiple primer pairs in one reaction can lead to spurious amplification products, e.g., primer dimers or non-specific target-primer binding [15]. Thus, an alternative strategy – one that can solve both challenges of assay design and multi-amplicon detection – is to use microfluidics to enable spatial segregation of the PCR mix and parallel amplification of each target into multiple compartments. Several fully-integrated multiplex PCR platforms (some of them relying on microfluidics technologies) have been developed, including QIAstat-Dx (Qiagen) [16], Verigene (Luminex) [17], xTAG (Luminex) [18], FilmArray (bioMérieux) [19], ePlex (GenMark Diagnostics) [20], Unyvero (OpGen) [21]. Importantly, these systems do not solely allow for PCR-based amplification of target DNA, but also integrate important pre-amplification steps, such as DNA extraction and purification, as well as post-amplification techniques for end-product identification, such as bead-based array [22], DNA hybridization and melting curve analysis [19]. Some of these technologies have been cleared by the US Food and Drug Administration (FDA), and are being used increasingly in the clinical setting, especially in clinical microbiology [24–26] (Fig. 1C). However, even if their impact on antibiotic stewardship is starting to be documented in large prospective trials [27], these commercially available PCR platforms used in syndromic testing still suffer from important limitations, including their price and relatively low throughput (Table 1).

Parallel amplification also forms the rationale behind digital PCR (dPCR; Fig. 1A). Here, the PCR mix is split into thousands of partitions for simultaneous amplification, but contrarily to the microfluidics platform described above, in multiplex dPCR all targets and all primer pairs are present in the partitions [28]. The partitions can be created using a number of different mechanisms, such as emulsified microdroplets suspended in oil (droplet digital PCR, ddPCR), microwells, or microfluidic valving. Amplification of target sequences can be detected by endpoint fluorescence, and some machines also provide real-time fluorescence data [29,30]. Importantly, dPCR enables precise quantification of targets by calculating the ratio of positive partitions (presence of fluorescence) over the total number of partitions. Even though dPCR has the advantage to yield rich data sets containing thousands of curves in each run, dPCR data also show much wider variability. Furthermore, dPCR remains expensive and low throughput, which has limited its use mostly to research purposes, and much less commonly to clinical applications [31].

## 3 Melting curve analysis

**Fig. 2:**
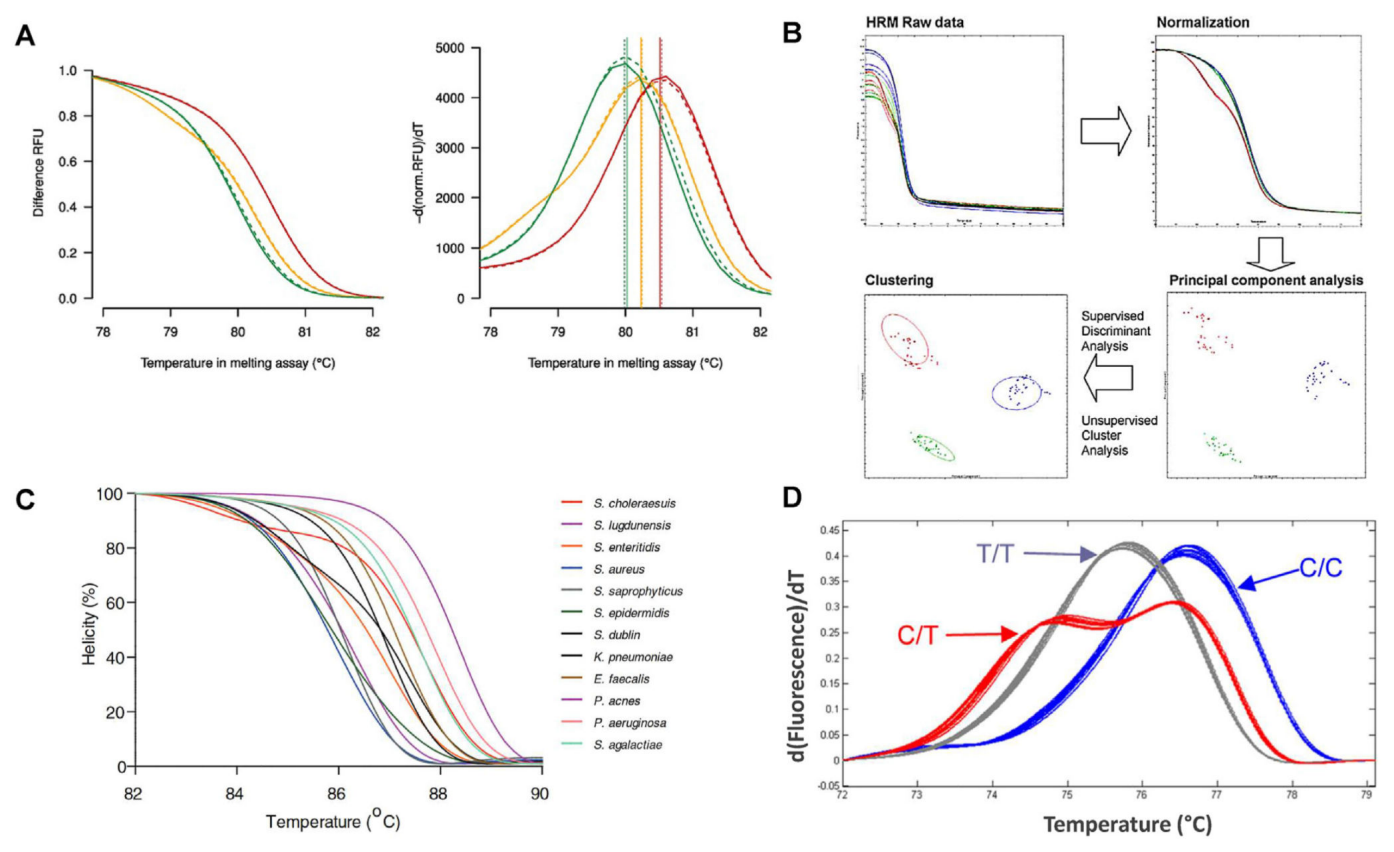
Melting curve analysis A. Typical melting curve (left plot) and its first-order derivative (right plot). Vertical dotted lines on the right plot indicate the T_m_ (copied from Ref. [82] under a Creative Commons license). B. Example of data analytics pipeline using the melting curve (adapted from Ref. [44]). C. Example showing how HR-MCA can be used for single-tube single-channel multiplex PCR in diagnostic microbiology (from Ref. [39]). D. Example showing that HR-MCA has enough resolution for SNP detection (from Ref. [35]).

During progressive and controlled heating of the products of an amplification reaction in the presence of fluorescent dyes, denaturation of double-stranded DNA into single strands generates a characteristic loss-of-fluorescence curve called the melting curve (MC, Fig. 2A) [32]. Its features are determined by the thermodynamic characteristics of the reaction, mainly related to the sequence of PCR products. More precisely, the energy required to break hydrogen bonds between two strands of DNA is dependent on their length, GC content (because G-C base pairings have 3 hydrogen bonds between them, while A-T base pairs only have 2) and their complementarity. This explains how the melting curve provides a unique DNA sequence signature that is useful for target identification, and forms the basis of MC analysis (MCA). Often, it is the first derivative of the MC that is computed, the peak of which (T_m_, for melting temperature) corresponds to the temperature at which 50% of the DNA molecules are double-stranded [32] (Fig. 2A). Generating a MC on an instrument that can control temperature and record fluorescence at each step with high precision enables high-resolution (HR) MCA, a process that has sufficient resolution to differentiate two amplicons that differ only by a single nucleotide polymorphism (SNP) [33], paving the way for using HR-MCA for genotyping applications (Fig. 2D) [34,35]. HR-MCA is particularly suited for multiplexing in clinical microbiology applications, where the number of potential targets is often high. For instance, it has been used to detect influenza and coronaviruses in single-channel mPCR assays [36,37]. It has been coupled with a PCR targeting highly variable regions of the bacterial genome (mostly the 16S region of ribosomal DNA) to identify pathogenic bacteria in clinical samples (Fig. 2C) [38,39]. Finally, HR-MCA has also been used for genotypic detection of antimicrobial resistance genes, for instance in multiplex assays detecting point mutations associated with cephalosporin and azithromycin resistance in *Neisseria gonorrhoeae* [40]. HR-MCA has also been implemented in microfluidics-based and dPCR platforms and can be used concurrently with spatial segregation to increase multiplexing [41].

Successful implementation of MCA is influenced by the analytical process used to extract information from MCs [42]. The signal processing pipeline has seen gradual improvement over the years, and generally includes several steps, including: background fluorescence subtraction and normalization; curve overlay, a “temperature shifting” of curves that allows to correct for minor temperature errors between samples and runs; variant clustering, using hierarchical clustering algorithms; computation of difference plots, the fluorescence in each variant cluster being subtracted from the average fluorescence of a reference cluster; computation of negative first derivative plots of normalized melting data using SavitzkyeGolay polynomial estimation (Fig. 2B) [43,44]. Signal processing techniques can be used to cluster MCs related to identical amplicons together, but for high-throughput labelling of large datasets of unknown samples, which implies matching each MC against a database of previously identified ones, methods beyond visual inspection or the clustering function included in the instrument software are needed. Recently, several ML algorithms have been successfully employed to carry out this classification problem. Athamanolap et al. generated MCs related to a fragment of the capsule polysaccharide synthesis (cps) gene locus of 92 serotypes of *S. pneumonia in silico* [45] and trained an ensemble of linear kernel support vector machine algorithm (SVM), resulting in an average classification accuracy of 99.9% [46]. *In vitro* verification of the algorithm was obtained using sequence variants of a cancer-related gene and demonstrated 100% accuracy with 3 training curves per sequence variant. In a following paper, the same team generated an experimental library of long amplicons (>1000 bp) MCs related to the 16S gene of 37 microorganisms and trained a nested SVM classifier, obtaining high accuracy with bacterial isolates but a limited classification performance on clinical samples [5]. Finally, convolutional neural networks have been used for classification of HR-MC data converted into images through the use of recurrence plots [47].

## 4 Amplification curve analysis

**Fig. 3:**
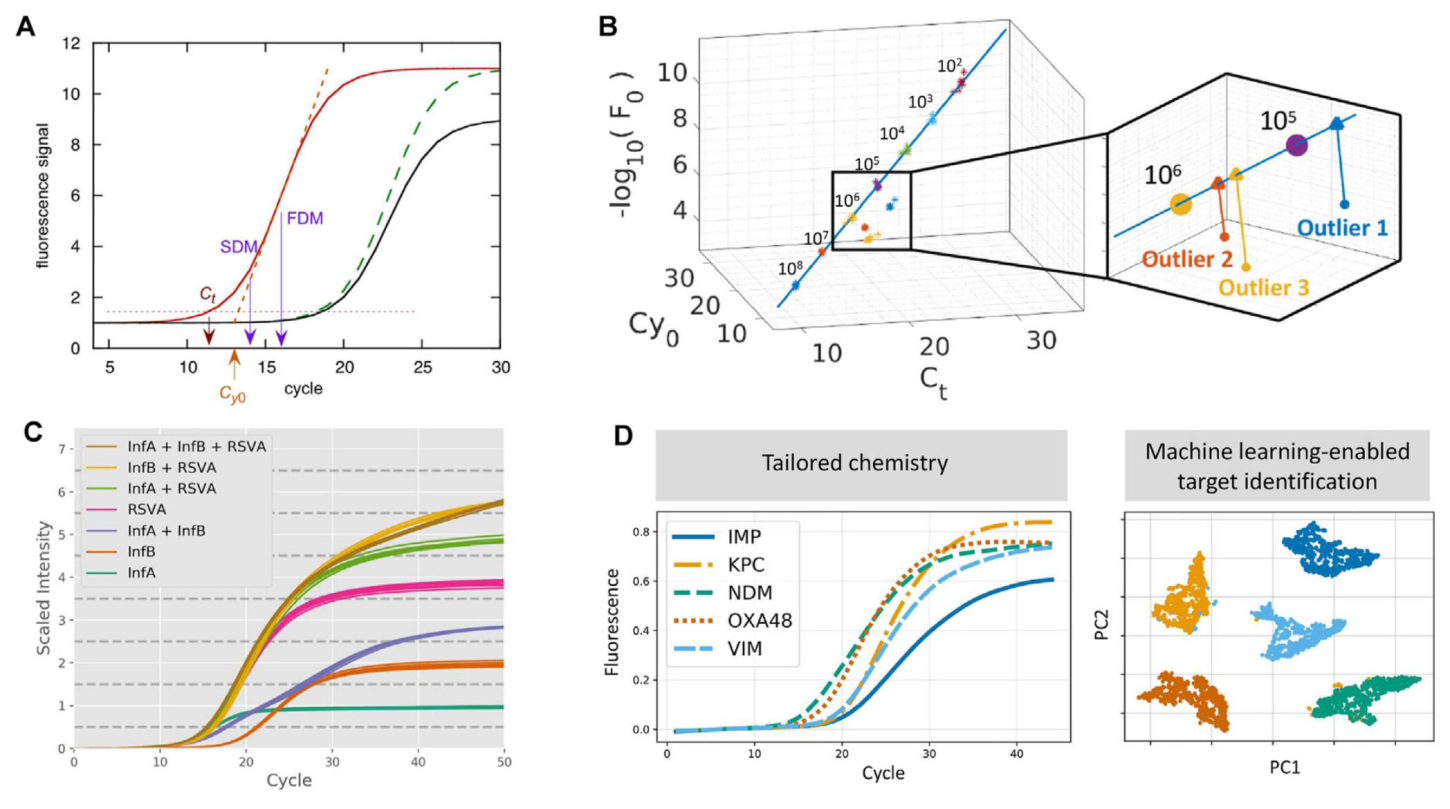
Amplification curve analysis A. Synthetic amplification curves (*n* ¼ 3) generated using a 4-parameter sigmoid equation, and 4 common location indices: C_t_, Cy_0_, FDM and SDM. Changing the values of parameters results in changing the location of the exponential phase, the slope of the linear phase, and the plateau (from Ref. [56]). B. A multidimensional standard curve (MSC) is constructed using C_t_, Cy_0_, and -log_10_(F_0_) with concentration values ranging from 10^2^ to 10^8^ of a synthetic DNA target. The right panel shows a zoomed region of the feature space and indicates the projection of outliers onto the MSC (copied from Ref. [58] without modification under Creative Commons CC-BY Usage Agreement). C. Final Fluorescence Intensity (FFI) modulation using TaqMan probes in one channel. This figure illustrates that FFI modulation can resolve co-infection cases if the concentrations of probes and primers are carefully chosen (42 nasopharyngeal samples spiked with 3 viral targets, 100% accuracy) (copied from Ref. [59] without modification). D. The core concepts of amplification curve analysis (ACA): careful assay design (“tailored chemistry”) enables to obtain amplification curves with specific features. These features can be extracted through machine learning and then used for target identification in a single-well, single-channel multiplex assay (adapted from Ref. [64]).

In a quantitative singleplex PCR assay using intercalating dyes, the level of fluorescence recorded at each cycle – which is related to the total amount of amplified DNA – enables plotting of a typical sigmoid-shaped curve, the amplification curve (AC; Fig. 3A) [11]. The AC typically has a baseline phase, where the number of amplified molecules is too low and the fluorescence remains below the limit of detection; a second phase of exponential growth, followed by a linear transitional phase; finally the amount of newly produced DNA molecules falls as limiting reagents (e.g., primers) get depleted and the activity of the polymerase decreases, leading to a plateau [48,49]. Thus, the shape of the AC reflects the dynamics of the amplification process, determined by characteristics of the PCR machine, initial concentrations of DNA molecules and reagents, the activity of the polymerase, the presence of inhibitors, and more generally the laws of thermodynamics that govern the interactions of all DNA molecules in the tube [50,51]. The AC has traditionally been used mainly to infer information related to the concentration of templates at the start of PCR, which forms the basis for its use in qPCR [51]. It is only recently that the AC has been successfully used to infer template sequence in multiplex assays.

It is beyond the scope of this review to provide a detailed analysis of existing algorithms that use the AC to infer quantitative information about template concentration, and the interested reader is directed to Refs. [51–53]. However, these methods share several key steps that we find interesting to discuss. Following background fluorescence removal and normalization, curves are usually fitted to a mathematically-defined ‘S’-shaped function linking cycle to fluorescence, most commonly using a 4-, 5- or 6- parameter sigmoid function [54,55]. The final step involves identifying a location parameter, usually the cycle value associated with a specific phase of the reaction: the commonly used cycle threshold (C_t_) value is the cycle when the fluorescence of PCR products reaches a specified threshold level; the first- and second-derivative maxima (FDM and SDM) are the cycle values where those derivatives reach their maxima; C_y0_ is the intersection of a line tangent to the curve at the FDM with the baseline-subtracted signal level (Fig. 3A) [56].

Beyond evaluation of PCR efficiency and initial target quantity, little effort has been invested in using data analytics to extract information related to target sequence from ACs, as can be done through HR-MCA. However, HR-MCA is usually performed at the end of PCR, which requires additional cycling time; it is also limited to intercalating dyes and to instruments with accurate thermal control. These limitations have motivated the use of the AC for target identification. The first breakthrough came from the realization that, during one given amplification reaction, the above-described location parameters could be used simultaneously to define multi-dimensional standard curves (MSCs), thus leveraging multiple physical features of the reaction in a shared analytics framework (Fig. 3B) [57,58]. MSCs were used for reliably identifying non-specific amplification events (outlier removal) and improving DNA quantification.

To be able to use differences in shapes of ACs across different targets in single-channel mPCR, it is possible to design assays in such a way as to produce highly differentiable curves, for instance by using different concentrations of primers and probes. Because the plateau phase of the amplification reaction is attained when limiting reagents have been consumed, there is a direct relationship (albeit not always linear) between the initial concentration of primers (when using intercalating dyes) and probes (in probe-based assays) and the final fluorescence intensity (FFI). This was demonstrated in Ref. [59] and used to design a probe-based single-channel 3-plex assay to detect 3 viruses (influenza A and B, VRS) in spiked nasopharyngeal samples with 100% accuracy across 36 samples (Fig. 3C), and then extended to design a 3- channel, 9-plex assay obtaining 98.8% accuracy over 1030 samples [60]. Elegantly, setting up probe concentrations at multiples of 2 (e.g., x, 2x and 4x) results in every possible combination of targets having a unique total digested probe concentration, and thus a unique fluorescence level; this makes the assay robust to detect to co-amplification events.

Similarly, Lee et al. have devised a technique called MuDT (Multiple Detection Temperatures), which enables detection of 2 targets simultaneously in a single fluorescent channel by using only the amplification signal [61]. MuDT relies on the Tagging Oligonucleotide Cleavage and Extension (TOCE) technique, where indirect temperature-dependent signals are generated at each cycle through the use of two oligo probes, namely the Pitcher and the Catcher. By designing Extender and Catcher sequences with specific T_m_, this fluorescence signal can be measured during a temperature-specific detection phase at the end of each annealing step, enabling real-time duplex detection and quantification (using the C_t_ value) as well as resolution of co-amplification events.

In the two latter examples, by designing assays to modulate the FFI or to generate sequence-dependent signals that can be detected at specific temperatures, one can obtained original amplification curves that are easily differentiated using simple data analytics techniques. This illustrates the important concept that mPCR assays where target identification is supported by ML algorithms can only work optimally if ML is coupled with tailored chemistry: the more assay designers can do to modulate the shape of ACs using biochemistry, the less sophisticated the data analytics process needs be.

**Fig. 4:**
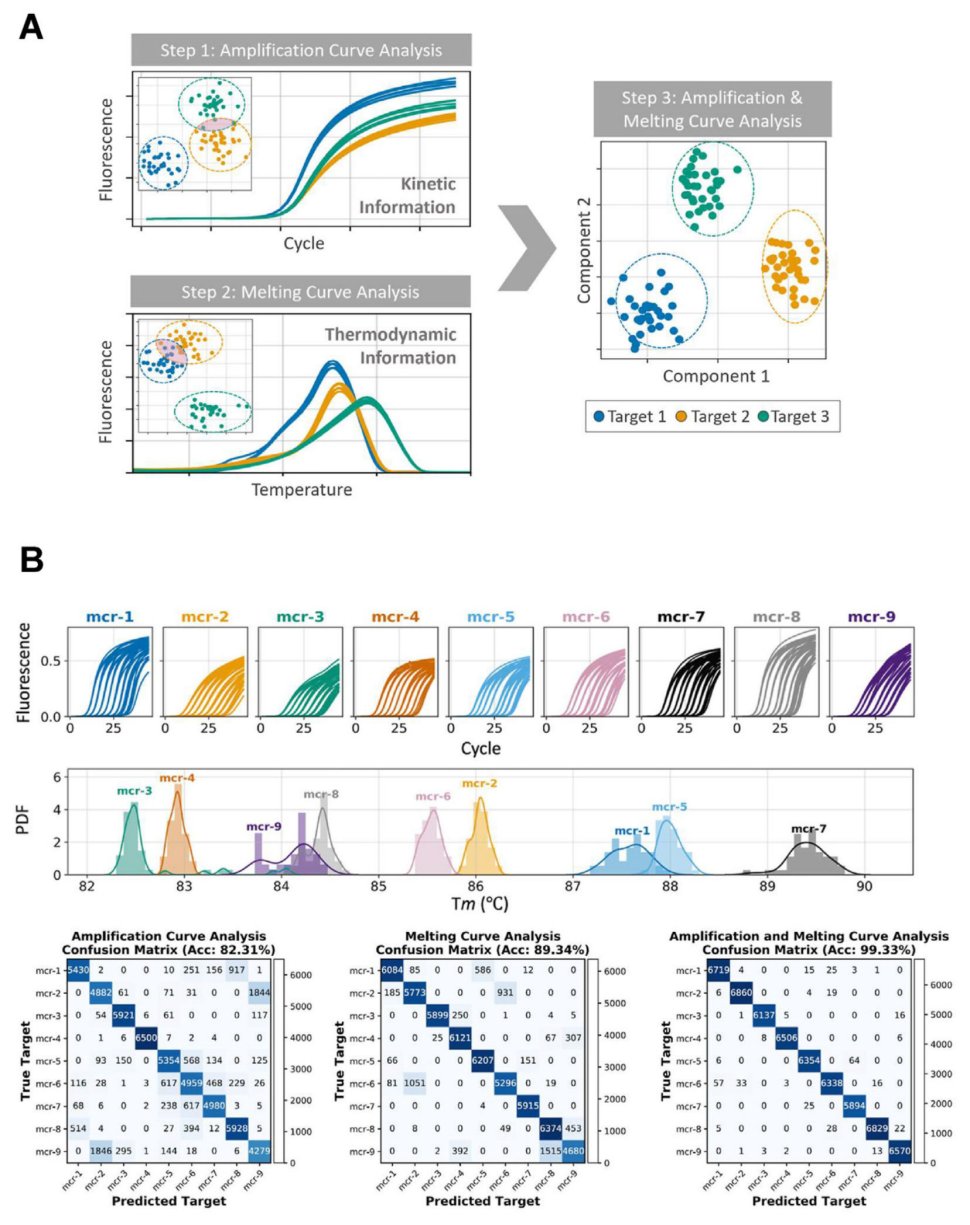
Amplification and melting curve analysis A. Schematic of the combined use of amplification and melting curves for target identification in mPCR. B. Example of application of amplification and melting curve analysis (AMCA). In Ref. [63], a 9-plex single-channel (intercalating dyes) multiplex digital PCR assay was developed to detect mobilized colistin resistance (mcr) genes, reaching classification accuracy of 99.33 ± 0.13% by combined analysis of ACs and MCs. As exemplified, non-redundant information can be extracted from ACs and MCs: overlapping MCs (e.g., mcr-8/mcr-9 and mcr-1/mcr-5) negatively impact on classification accuracy based on MCA alone, and those cases can be resolved through ACA (Fig. 4A and B both adapted from Ref. [63], copyright 2020 American Chemical Society).

Building up on the MSC framework, our group has worked towards applying advanced ML techniques to use ACs for accurate target identification in mPCR, an approach called amplification curve analysis (ACA; Fig. 3D). To obtain high classification accuracy, most ML algorithms need to be initially trained on large amounts of data; consequently, ACA has thus far been successfully applied to increase multiplexing capabilities of existing dPCR platforms using dye-based chemistries. In Ref. [62], a 3-plex assay to identify 3 distinct carbapenemase genes attained an accuracy ranging from 92.9% (when all 3 targets were combined in one assay, including co-amplification events) to 99.1% (with single targets). In Ref. [63], ACA was used in a 9-plex assay to detect mobilized colistin resistant genes, resulting in a classification accuracy of >99% (Fig. 4). In this paper, ACA alone did not reach optimal accuracy but was coupled with MCA to resolve cases where the MCA classifier could not differentiate two target sequences with overlapping T_m_. This combined use of melting and amplification curve analysis (AMCA) was further applied on bacterial isolates in a 5-plex assay with >99% accuracy to detect carbapenemase genes, demonstrating the robustness of the technique and its potential for clinical applications [64]. To further improve classification accuracy, a post-processing outlier detection algorithm has been developed, which can identify and filter out nonspecific and low-efficient reactions from dPCR data using sigmoidal trends of ACs [65]. Finally, to enhance the transferability of ACA across platforms and sample types, we have implemented a transformer-based conditional domain adversarial network (T-CDAN) that can reliably eliminate distribution differences between data obtained on synthetic DNA and clinical isolates [66].

## 5 Data-driven technologies for multiplexing based on isothermal amplification chemistries

Novel NAAT chemistries sharing the common feature of being isothermal (i.e., performed at constant temperature) have been developed in the last decades. These methods have emerged as an alternative to PCR, avoiding the need of thermal cycling and therefore being ideal molecular methods for rapid and accurate point-of-care diagnostics [67,68]. Among all the isothermal amplification methods, the most commonly used include: loop-mediated isothermal amplification (LAMP) [69], helicase dependent amplification (HDA) [70], and recombinase polymerase amplification (RPA) [71]. In particular, LAMP has attracted a lot of attention in the last years and several techniques have been employed to multiplex targets using this isothermal chemistry. LAMP is based on refolding, generating self-priming structures that can be used as new templates for amplification, which requires a DNA polymerase with strand displacement activity.

In order to increase the throughput of LAMP and to maximize the use of the amplification data, single-tube single-channel multiplexing in LAMP has mainly been achieved through the application of MCA. Because LAMP end-products are stem-loop DNA structures with several repeats of the target sequence whose exact nature and relative concentrations are difficult to predict, applying MCA and ACA for target identification in multiplex LAMP could in theory appear challenging. However, several groups have shown that under given experimental conditions – i.e., target and primer sequences, reagents and polymerase – melting of LAMP end-products gives uniform T_m_ values, both in bulk-based and single-molecule assays [72,73]. For instance, in a multiplex LAMP assay targeting influenza A and B, the mean T_m_ were 86.6°C for influenza A and 82.7°C for influenza B, allowing for their classification based on MCA [74]. Another work integrated HR-MC pattern recognition using principal component analysis of the data obtained with LAMP for the discrimination of three targets with a minimum T_m_ difference of 1.29 ± 0.04°C [75].

Finally, target identification in multiplex LAMP assays can also be achieving using ACA. Indeed, we have recently combined five digital LAMP assays in a single-tube single-channel (using the non-specific dye EvaGreen) multiplex assay targeting respiratory pathogens, where target classification was achieved through ACA with a global accuracy of 91.33% [76].

## 6 *In silico* prediction of amplification and melting curves: knowledge-based and data-driven solutions for assay design

Accurate prediction of DNA secondary structure and hybridization, which results from strong non-covalent interactions between complementary Watson-Crick base pairs, has been a topic of intense research for decades. In its most simple form, DNA secondary structure prediction compares the free energy changes (ΔG^0^) predicted for the formation of all possible secondary structures: since the equilibrium constant $K=\frac{\left[B\right]}{\left[A\right]}$ for a conformational change *A* → *B* is equal to $e^{\frac{-\Delta G^{0}}{RT}}$ (where R is the gas constant [equal to 8.314 J K^−1^ mol^−1^] and T is the temperature in Kelvin), the conformation with the lowest free energy dominates at equilibrium. To compute ΔG^0^ (or T_m_, equivalently) for a full DNA sequence, the nearest-neighbor (NN) approach is based on the assumption that the interaction between complementary base pairs depends on the neighboring base pairs. For each NN pair, it can be calculated as ΔG^0^ = H − TS, where H is the enthalpy, T is the temperature and S is the entropy. Entropy and enthalpy values for each NN base pairs have been obtained experimentally, and can be accessed in several databases of thermodynamic parameters [77,78]. Application of thermodynamic tables to accurately predict hybridization of long (>100 nucleotides) sequences was made possible by the development of powerful dynamic programming algorithms. Furthermore, to increase prediction accuracy, corrections to the simplest NN model have been calculated, including internal and terminal mismatches, dangling ends, hairpins, bulges, internal and multi-branched loops. Among software including thermodynamic databases and using functional programming to predict DNA secondary structures, melting temperatures (and for some [45], equilibrium melting profiles over a range of temperatures) are MELTING [6], uMELT [45], dnaMATE [7], DINAMelt [8], and NUPACK [9].

Prediction of ACs has received much less attention, probably because the use of ACs for target identification in mPCR is in its infancy compared to MCA. However, the same thermodynamic information can be used to characterize the kinetics of the annealing reaction, and thus to compute how the concentration of templates, primers and amplicons evolve at each cycle. Other parameters such as polymerase efficiency and platform-specific settings can further be included in the simulation, leading to *in silico* predictions of ACs.

**Fig. 5:**
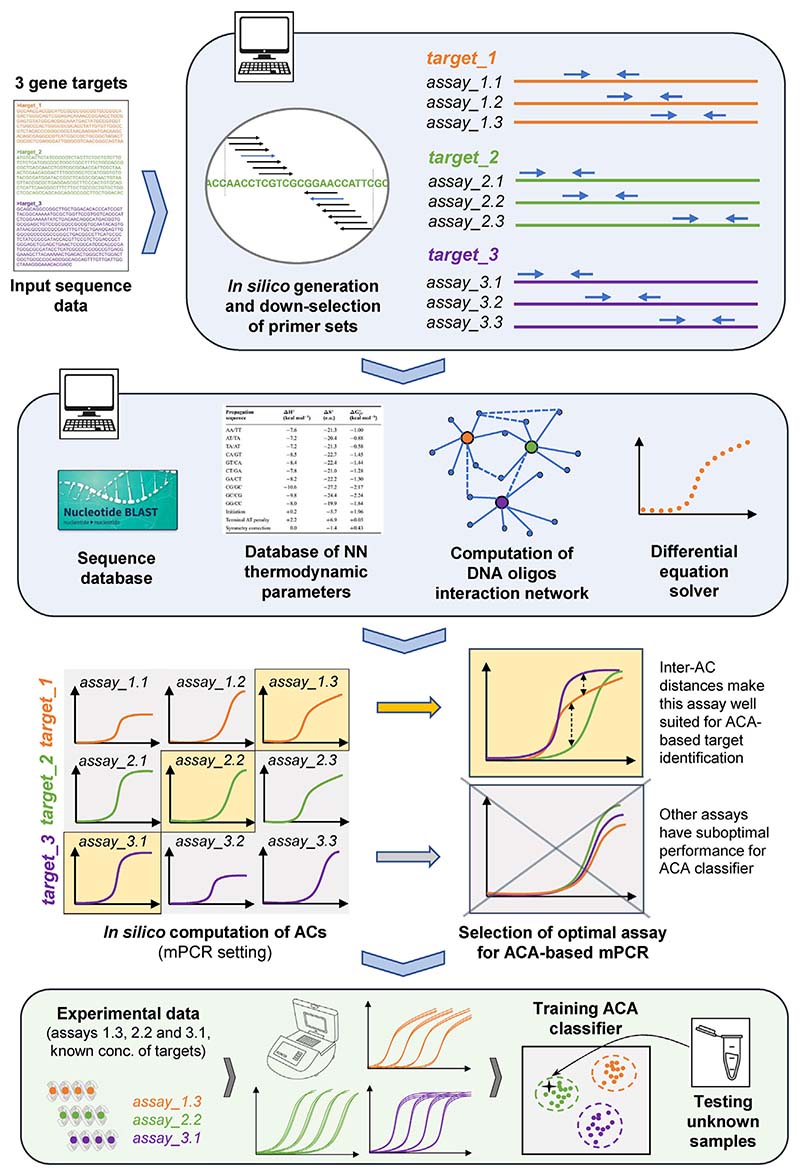
*In silico* prediction of amplification reactions output This figure presents an integrated framework linking *in silico* prediction of PCR output with machine learning-enabled target identification using amplification curves (ACA). In this example, we aim to design an optimal 3-plex assay in one channel using ACA. The first computational step consists in identifying primers fulfilling pre-specified criteria (e.g., T_m_, GC content, alignment, etc.). This leads to the down-selection of a set of possible assays for each target. The second *in silico* step uses sequence and thermodynamics databases to calculate free energy levels for all molecules in the system and compute their interaction network. This is used to predict how concentrations of all molecules evolve at each cycle (by solving a system of differential equations). Among the 27 multiplex assays that can be implemented with *m* = 3 designs for *n* = 3 targets (*m^n^*=27 possibilities), we select the one(s) where ACs are easily differentiated based on an inter-AC distance metric. This optimal ACA-based mPCR assay can then be implemented experimentally: PCR experiments with known concentrations of targets (synthetic DNA) are used to train an ACA classifier. This classifier can be in turn used for target identification of unknown samples. Importantly, a similar strategy can be implemented using the MC, as we expect AMCA-based PCR assays to have higher performance than assays based only on one type of curve. See Refs. [79,80] and for further detail on methodology and implementation.

As previously mentioned, in a data-driven framework, algorithms developed for target identification in mPCR must be coupled with tailored chemistry for optimal performance. In a multiplex assay designed to detect *n* targets, with *m* possible ways to design primers for each target, there will be *m^n^* possible combinations of primer pairs, making it impossible to test experimentally all such possible combinations. Thus, *in silico* simulations that can compute the free energy levels associated with the interaction of all molecules (including all primers and all potential targets) in an assay, predict the evolution of concentrations during thermal cycling and simulate ACs and MCs for end-products could become extremely helpful to narrow down the set of optimal primers to test experimentally (Fig. 5). Equipped with such software – functioning as a digital twin of a multiplex amplification reaction – assay designers willing to leverage the data-driven framework could test among all possible combinations of primer pairs and select those having minimal non-specific primer binding and resulting in the most easily distinguishable amplification and melting curves. In line of this, we have recently developed the SmartPlexer, an algorithm that uses experimental singleplex PCR data to compute distance measurements between ACs and rank all possible multiplex combinations from high to low inter-curve similarity values [79]. In this paper, SmartPlexer was successfully implemented to test all possible combinations (*n* = 4608) in a 7-plex TaqMan-based assay using ACA to identify 7 common pathogens responsible for respiratory tract infections. Of note, finding optimal primer sets (those that minimize the probability of primer dimers) can also be carried out *in silico* through the use of stochastic algorithms [80].

The most promising approach might be in combining both computational and experimental inputs for AC and MC prediction. In this framework, a physical (knowledge-based) model based on thermodynamic data would be used as a backbone to make predictions that would be in agreement with physical laws, but would suffer from approximations and would not capture platform-specific parameters, as well as inherent experimental variability. This physical model would be coupled with a data-driven model meant to simulate this run-to-run or well-to-well experimental noise, and developed using machine learning and a limited set of experimental data. An example of such approach comes from Langouche et al. [5], where *in silico* melting curves for the 16S rRNA gene of ten different bacteria are generated using uMELT and coupled with a noise envelope computed from experimental data using dynamic time warping.

Another interesting field of application of digital solutions lies in the detection of non-specific interactions, such as primer dimers, which are an important concern in mPCR. First, the free energy associated with a primer dimer can be computed using the same databases that are used to predict specific interactions, which implies that these non-specific reactions could be predicted (at least in part) and included in the reaction interaction network. Second, even if the reactions cannot be entirely predicted, their stochastic nature tends to increase the noise around the total signal of a given amplification reaction, which could result in less accurate machine learning-based classifiers for target identification. However, in a framework associating a physical model based on thermodynamic information and extraction of features related to experimental variability through machine learning, we envision that these non-specific amplification products could accurately be detected using software solutions.

Lastly, another critical step where the use of digital methodologies could be promising tools is the design of LAMP assays, which are not trivial to implement. Recently, Xu et al. explored the relationship between hairpin and amplicon-mediated nucleic acid amplification using reaction graph abstractions [81]. This model describes isothermal amplification mechanisms, requiring as input the specific target sequence and providing as output a set of assays and a proposed isothermal amplification process. The authors propose two strategies: generic tail strategy and progressive modeling. The first relies on hairpins and universal sequences aimed to be applied for the detection of short targets such as miRNA, and the latter focuses on using intermediate structures as triggers for different reaction pathways. This is a promising tool which could be further adapted to specific assay requirements, reducing the current challenges in assay design.

## 7 Conclusion

Data-driven technologies, specifically machine learning algorithms, have started to be coupled with existing PCR platforms and chemistries to enhance their multiplexing capabilities. These technological advances offer the prospect to develop inexpensive, high-throughput and robust assays that could fulfil the growing need to detect and quantify multiple nucleic acid sequences in one single reaction. Because they can be applied to any nucleic acid sequence, their potential impact in biotechnology, biomedical research and diagnostics is expected to extend across many fields of application. Furthermore, we envision that improvements in computer simulation will make it possible to streamline the design of data-driven mPCR assays through *in silico* primer optimization and prediction of reaction outcome, leading to optimal detection accuracy. We envision that concerted efforts by academic investigators and industry scientists will be needed to advance these technologies to a level of maturity, robustness and versatility that would make them suitable to be integrated in next-generation diagnostic platforms.

## Figures and Tables

**Table 1:** Limitations of existing multiplex real-time PCR technologies.

Single-well technologies	Spatial multiplexing (microfluidics-based) technologies
Low throughput: the number of targets that can be identified simultaneously in one sample is limited by the number of channels available in the PCR machine (up to 6 channels). Complexity of assay design and chemistry optimization: longer development time, possible incompatibility of primers and probes and multiplex-specific problems, such as competition between the amplification modules. Limited to expensive chemistries (e.g., probes) or need for post-PCR processes.	Sample preservation: splitting up samples in multiple compartments can be problematic is sample volume is limited. Compromise between reagent costs and reaction volume.
	Requirement for dedicated platforms and cartridges; longer hardware development time (to reconfigure specifications). Expensive equipment. Mostly limited to specialized laboratory environment.

## Data Availability

No data was used for the research described in the article.

## Abbreviations

AC: amplification curve
ACA: amplification curve analysis
AMCA: amplification and melting curve analysis
FDM: first derivative maxima (of amplification curve)
FFI: final fluorescence intensity
HR-MCA: high-resolution melting curve analysis
LAMP: loop-mediated isothermal amplification
MC: melting curve
MCA: melting curve analysis
MSC: multi-dimensional standard curve
ML: machine learning
NAATs: nucleic acid amplification tests
NN: nearest-neighbor
PCR: polymerase chain reaction
dPCR: digital PCR
ddPCR: droplet digital PCR
mPCR: multiplex PCR
qPCR: quantitative PCR
rtPCR: real-time PCR
SDM: second derivative maxima (of amplification curve)
SVM: support vector machine

## References

[1] Shin DJ, Andini N, Hsieh K, Yang S, Wang T-H (2019). Emerging analytical techniques for rapid pathogen identification and susceptibility testing. Annu Rev Anal Chem.

[2] Zhu H, Zhang H, Xu Y, Laššáková S, Korabečná M, Neužil P (2020). PCR past, presa, M. Korabea, P. Neusent and future. Biotechniques.

[3] Kubista M, Andrade JM, Bengtsson M, Forootan A, Jonák J, Lind K (2006). The real-time polymerase chain reaction. Mol Aspect Med.

[4] Gray J, Coupland LJ (2014). The increasing application of multiplex nucleic acid detection tests to the diagnosis of syndromic infections. Epidemiol Infect.

[5] Langouche L, Aralar A, Sinha M, Lawrence SM, Fraley SI, Coleman TP (2020). Data-driven noise modeling of digital DNA melting analysis enables prediction of sequence discriminating power. Bioinformatics.

[6] Dumousseau M, Rodriguez N, Juty N, Novère NL (2012). MELTING, a flexible platform to predict the melting temperatures of nucleic acids. BMC Bioinf.

[7] Panjkovich A, Norambuena T, Melo F (2005). dnaMATE: a consensus melting temperature prediction server for short DNA sequences. Nucleic Acids Res.

[8] Markham NR, Zuker M (2005). DINAMelt web server for nucleic acid melting prediction. Nucleic Acids Res.

[9] Fornace ME, Porubsky NJ, Pierce NA (2020). A unified dynamic programming framework for the analysis of interacting nucleic acid strands: enhanced models, scalability, and speed. ACS Synth Biol.

[10] Wittwer CT, Herrmann MG, Gundry CN, Elenitoba-Johnson KSJ (2001). Real-time multiplex PCR assays. Methods.

[11] Heid CA, Stevens J, Livak KJ, Williams PM (1996). Real time quantitative PCR. Genome Res.

[12] Mhlanga MM, Malmberg L (2001). Using molecular beacons to detect single-nucleotide polymorphisms with real-time PCR. Methods.

[13] Whitcombe D, Theaker J, Guy SP, Brown T, Little S (1999). Detection of PCR products using self-probing amplicons and fluorescence. Nat Biotechnol.

[14] Nazarenko IA, Bhatnagar SK, Hohman RJ (1997). A closed tube format for amplification and detection of DNA based on energy transfer. Nucleic Acids Res.

[15] Markoulatos P, Siafakas N, Moncany M (2002). Multiplex polymerase chain reaction: a practical approach. J Clin Lab Anal.

[16] Boers SA, Melchers WJG, Peters CJA, Toonen M, McHugh MP, Templeton KE (2020). Multicenter evaluation of QIAstat-dx respiratory panel V2 for detection of viral and bacterial respiratory pathogens. J Clin Microbiol.

[17] Kosai K, Suzuki H, Tamai K, Okada Y, Akamatsu N, Ueda A (2021). Multicenter evaluation of Verigene enteric pathogens nucleic acid test for detection of gastrointestinal pathogens. Sci Rep Nat Publ Group.

[18] Pabbaraju K, Wong S, Tokaryk KL, Fonseca K, Drews SJ (2011). Comparison of the luminex xTAG respiratory viral panel with xTAG respiratory viral panel fast for diagnosis of respiratory virus infections. J Clin Microbiol Am Soc Microbiol.

[19] Holma T, Torvikoski J, Friberg N, Nevalainen A, Tarkka E, Antikainen J (2022). Rapid molecular detection of pathogenic microorganisms and antimicrobial resistance markers in blood cultures: evaluation and utility of the next-generation FilmArray Blood Culture Identification 2 panel. Eur J Clin Microbiol Infect Dis.

[20] Jarrett J, Uhteg K, Forman MS, Hanlon A, Vargas C, Carroll KC (2021). Clinical performance of the GenMark Dx ePlex respiratory pathogen panels for upper and lower respiratory tract infections. J Clin Virol.

[21] Klein M, Bacher J, Barth S, Atrzadeh F, Siebenhaller K, Ferreira I (2021). Multicenter evaluation of the Unyvero platform for testing bronchoalveolar lavage fluid. J Clin Microbiol Am Soc Microbiol.

[22] Zou S, Han J, Wen L, Liu Y, Cronin K, Lum SH (2007). Human influenza A virus (H5N1) detection by a novel multiplex PCR typing method. J Clin Microbiol.

[24] Huang H-S, Tsai C-L, Chang J, Hsu T-C, Lin S, Lee C-C (2018). Multiplex PCR system for the rapid diagnosis of respiratory virus infection: systematic review and meta-analysis. Clin Microbiol Infect.

[25] Caliendo AM (2011). Multiplex PCR and emerging technologies for the detection of respiratory pathogens. Clin Infect Dis.

[26] Otoo JA, Schlappi TS (2022). REASSURED Multiplex Diagnostics: A Critical Review and Forecast. Biosensors.

[27] Darie AM, Khanna N, Jahn K, Osthoff M, Bassetti S, Osthoff M (2022). Fast multiplex bacterial PCR of bronchoalveolar lavage for antibiotic stewardship in hospitalised patients with pneumonia at risk of Gram-negative bacterial infection (Flagship II): a multicentre, randomised controlled trial. Lancet Respir Med.

[28] Quan P-L, Sauzade M, Brouzes E (2018). dPCR: a technology review. Sensors.

[29] Tan LL, Loganathan N, Agarwalla S, Yang C, Yuan W, Zeng J (2022). Current commercial dPCR platforms: technology and market review, Taylor & Francis. Crit Rev Biotechnol.

[30] Witters D, Sun B, Begolo S, Rodriguez-Manzano J, Robles W, Ismagilov FR (2014). Digital biology and chemistry, Lab on a Chip. Royal Soc Chem.

[31] Ganova M, Zhang H, Zhu H, Korabea M, Neucnzil P (2021). Multiplexed digital polymerase chain reaction as a powerful diagnostic tool, Biosens. Bioelectron.

[32] Montgomery JL, Sanford LN, Wittwer CT (2010). High-resolution DNA melting analysis in clinical research and diagnostics. Expert Rev Mol Diagn.

[33] Reed GH, Wittwer CT (2004). Sensitivity and specificity of single-nucleotide polymorphism scanning by high-resolution melting analysis. Clin Chem.

[34] Liew M, Pryor R, Palais R, Meadows C, Erali M, Lyon E (2004). Genotyping of single-nucleotide polymorphisms by high-resolution melting of small amplicons. Clin Chem.

[35] Li F, Niu B, Huang Y, Meng Z (2012). Application of High-Resolution DNA Melting for Genotyping in Lepidopteran Non-model Species: Ostrinia Furnacalis (Crambidae). PLOS ONE.

[36] Ahberg CD, Manz A, Neuzil P (2015). Single fluorescence channel-based multiplex detection of avian influenza virus by quantitative PCR with intercalating dye. Sci Rep.

[37] Wan Z, Zhang Y, He Z, Liu J, Lan K, Hu Y (2016). A melting curve-based multiplex RT-qPCR assay for simultaneous detection of four human corona-viruses. Int J Mol Sci.

[38] Yang S, Ramachandran P, Rothman R, Hsieh Y-H, Hardick A, Won H (2009). Rapid identification of biothreat and other clinically relevant bacterial species by use of universal PCR coupled with high-resolution melting analysis. J Clin Microbiol.

[39] Fraley SI, Hardick J, Masek BJ, Athamanolap P, Rothman RE, Gaydos CA (2013). Universal digital high-resolution melt: a novel approach to broad-based profiling of heterogeneous biological samples. Nucleic Acids Res.

[40] Li Y, Zhang L, Xiu L, Wang D, Zeng Y, Wang F (2022). A multiplex molecular assay for detection of six penA codons to predict decreased susceptibility to cephalosporins in Neisseria gonorrhoeae. Antimicrob Agents Chemother.

[41] O’Keefe CM, Pisanic TR, Zec H, Overman MJ, Herman JG, Wang T-H (2018). Facile profiling of molecular heterogeneity by microfluidic digital melt. Sci Adv Am Assoc Adv Sci.

[42] Palais R, Wittwer CT (2009). Chapter 13 mathematical algorithms for high-resolution DNA melting analysis. Methods Enzymol.

[43] Montgomery J, Wittwer CT, Palais R, Zhou L (2007). Simultaneous mutation scanning and genotyping by high-resolution DNA melting analysis. Nat Protoc.

[44] Reja V, Kwok A, Stone G, Yang L, Missel A, Menzel C (2010). ScreenClust: advanced statistical software for supervised and unsupervised high resolution melting (HRM) analysis. Methods.

[45] Dwight Z, Palais R, Wittwer CT (2011). uMELT: prediction of high-resolution melting curves and dynamic melting profiles of PCR products in a rich web application. Bioinformatics.

[46] Athamanolap P, Parekh V, Fraley SI, Agarwal V, Shin DJ, Jacobs MA (2014). Trainable High Resolution Melt Curve Machine Learning Classifier for Large-Scale Reliable Genotyping of Sequence Variants. PLOS ONE.

[47] Ozkok FO, Celik M (2021). Convolutional neural network analysis of recurrence plots for high resolution melting classification. Comput Methods Progr Biomed.

[48] Liu W, Saint DA (2002). A new quantitative method of real time reverse transcription polymerase chain reaction assay based on simulation of polymerase chain reaction kinetics. Anal Biochem.

[49] Peirson SN, Butler JN, Foster RG (2003). Experimental validation of novel and conventional approaches to quantitative real-time PCR data analysis. Nucleic Acids Res.

[50] Gevertz JL, Dunn SM, Roth CM (2005). Mathematical model of real-time PCR kinetics. Biotechnol Bioeng.

[51] Rutledge RG, Côtée C (2003). Mathematics of quantitative kinetic PCR and the application of standard curves. Nucleic Acids Res.

[52] Ruijter JM, Pfaffl MW, Zhao S, Spiess AN, Boggy G, Blom J (2013). Evaluation of qPCR curve analysis methods for reliable biomarker discovery: bias, resolution, precision, and implications. Methods.

[53] Zhang Y, Li H, Shang S, Meng S, Lin T, Zhang Y (2021). Evaluation validation of a qPCR curve analysis method and conventional approaches. BMC Genom.

[54] Rutledge RG (2004). Sigmoidal curve-fitting redefines quantitative real-time PCR with the prospective of developing automated high-throughput applications. Nucleic Acids Res.

[55] Spiess A-N, Feig C, Ritz C (2008). Highly accurate sigmoidal fitting of real-time PCR data by introducing a parameter for asymmetry. BMC Bioinf.

[56] Tellinghuisen J, Spiess A-N (2014). Comparing real-time quantitative polymerase chain reaction analysis methods for precision, linearity, and accuracy of estimating amplification efficiency. Anal Biochem.

[57] Rodriguez-Manzano J, Moniri A, Malpartida-Cardenas K, Dronavalli J, Davies F, Holmes A (2019). Simultaneous single-channel multiplexing and quantification of carbapenem-resistant genes using multidimensional standard curves. Anal Chem Am Chem Soc.

[58] Moniri A, Rodriguez-Manzano J, Malpartida-Cardenas K, Yu L-S, Didelot X, Holmes A (2019). Framework for DNA quantification and outlier detection using multidimensional standard curves. Anal Chem Am Chem Soc.

[59] Rajagopal A, Yurk D, Shin C, Menge K, Jacky L, Fraser S (2019). Significant expansion of real-time PCR multiplexing with traditional chemistries using amplitude modulation. Sci Rep.

[60] Jacky L, Yurk D, Alvarado J, Belitz P, Fathe K, MacDonald C (2021). Robust multichannel encoding for highly multiplexed quantitative PCR. Anal Chem Am Chem Soc.

[61] Lee Y-J, Kim D, Lee K, Chun J-Y (2014). Single-channel multiplexing without melting curve analysis in real-time PCR. Sci Rep.

[62] Moniri A, Miglietta L, Malpartida-Cardenas K, Pennisi I, Cacho-Soblechero M, Moser N (2020). Amplification curve analysis: data-driven multiplexing using real-time digital PCR. Anal Chem Am Chem Soc.

[63] Moniri A, Miglietta L, Holmes A, Georgiou P, Rodriguez-Manzano J (2020). High-level multiplexing in digital PCR with intercalating dyes by coupling real-time kinetics and melting curve analysis. Anal Chem Am Chem Soc.

[64] Miglietta L, Moniri A, Pennisi I, Malpartida-Cardenas K, Abbas H, Hill-Cawthorne K (2021). Coupling Machine Learning and High Throughput Multiplex Digital PCR Enables Accurate Detection of Carbapenem-Resistant Genes in Clinical Isolates. Front Mol Biosci.

[65] Miglietta L, Xu K, Chhaya P, Kreitmann L, Hill-Cawthorne K, Bolt F (2022). Adaptive filtering framework to remove nonspecific and low-efficiency reactions in multiplex digital PCR based on sigmoidal trends. Anal Chem.

[66] Mao Ye, Xu Ke, Miglietta Luca, Kreitmann Louis, Georgiou Pantelis, Holmes Alison Deep Domain Adaptation Enhances Amplification Curve Analysis for Single-Channel Multiplexing in Real-Time PCR.

[67] Glokler J, Lim TS, Ida J, Frohme M (2021). Isothermal ampli€fications - a comprehensive review on current methods. Crit Rev Biochem Mol Biol.

[68] Fakruddin M, Mannan KSB, Chowdhury A, Mazumdar RM, Hossain MN, Islam S (2013). Nucleic acid amplification: alternative methods of polymerase chain reaction. J Pharm BioAllied Sci.

[69] Notomi T, Okayama H, Masubuchi H, Yonekawa T, Watanabe K, Amino N (2000). Loop-mediated isothermal amplification of DNA. Nucleic Acids Res.

[70] Vincent M, Xu Y, Kong H (2004). Helicase-dependent isothermal DNA amplification. EMBO Rep.

[71] Piepenburg O, Williams CH, Stemple DL, Armes NA (2006). DNA detection using recombination proteins. PLOS Biol Public Library Sci.

[72] Tone K, Fujisaki R, Yamazaki T, Makimura K (2017). Enhancing melting curve analysis for the discrimination of loop-mediated isothermal amplification products from four pathogenic molds: use of inorganic pyrophosphatase and its effect in reducing the variance in melting temperature values. J Microbiol Methods.

[73] Rolando JC, Jue E, Barlow JT, Ismagilov RF (2020). Real-time kinetics and high-resolution melt curves in single-molecule digital LAMP to differentiate and study specific and non-specific amplification. Nucleic Acids Res.

[74] Mahony J, Chong S, Bulir D, Ruyter A, Mwawasi K, Waltho D (2013). Multiplex loop-mediated isothermal amplification (M-LAMP) assay for the detection of influenza A/H1, A/H3 and influenza B can provide a specimen-to-result diagnosis in 40 min with single genome copy sensitivity. J Clin Virol.

[75] Dong J, Xu Q, Li C-C, Zhang C-Y (2019). Single-color multiplexing by the integration of high-resolution melting pattern recognition with loop-mediated isothermal amplification. Chem Commun.

[76] Malpartida-Cardenas K, Miglietta L, Peng T, Moniri A, Holmes A, Georgiou P (2022). Single-channel digital LAMP multiplexing using amplification curve analysis. Sensor Diagnostics.

[77] SantaLucia J, Hicks D (2004). The thermodynamics of DNA structural motifs. Annu Rev Biophys Biomol Struct.

[78] Turner DH, Mathews DH (2010). NNDB: the nearest neighbor parameter database for predicting stability of nucleic acid secondary structure. Nucleic Acids Res.

[79] (2022). Smart-Plexer: a breakthrough workflow for hybrid development of multiplex PCR assays. https://www.researchsquare.com.

[80] Xie NG, Wang MX, Song P, Mao S, Wang Y, Yang Y (2022). Designing highly multiplex PCR primer sets with simulated annealing design using dimer likelihood estimation (SADDLE). Nat Commun Nat Publ Group.

[81] Xu G, Reboud J, Guo Y, Yang H, Gu H, Fan C (2022). Programmable design of isothermal nucleic acid diagnostic assays through abstraction-based models. Nat Commun Nat Publ Group.

[82] Ashrafi R, Bruneaux M, Sundberg L-R, Pulkkinen K, Ketola T (2017). Application of high resolution melting assay (HRM) to study temperature-dependent intraspecific competition in a pathogenic bacterium. Sci Rep Nat Publ Group.

